# Estimation of measles risk using the World Health Organization Measles Programmatic Risk Assessment Tool, Iran

**DOI:** 10.1016/j.heliyon.2018.e00886

**Published:** 2018-11-01

**Authors:** Abolfazl Mohammadbeigi, Seyed Mohsen Zahraei, Azadeh Asgarian, Sima Afrashteh, Narges Mohammadsalehi, Salman Khazaei, Hossein Ansari

**Affiliations:** a Research Center for Environmental Pollutants, Qom University of Medical Sciences, Qom, Iran; b Centre for Communicable Diseases Control, Ministry of Health and Medical Education, Tehran, Iran; c Neurology and Neurosciences Research Center, Qom University of Medical Sciences, Qom, Iran; d MSc of Epidemiology, Bushehr University of Medical Sciences, Bushehr, Iran; e Department of Epidemiology, School of Public Health, Hamadan University of Medical Sciences, Hamadan, Iran; f Health Promotion Research Center, Department of Epidemiology and Biostatistics, Zahedan University of Medical Sciences, Zahedan, Iran

**Keywords:** Epidemiology, Public health, Infectious disease

## Abstract

**Introduction:**

Based on the World Health Organization (WHO) reports the EMRO countries did not reached to eradication of measles at 2010. This study aimed to estimate the risk of measles outbreak in different districts of Iran to identify high-risk areas based on WHO measles programmatic risk assessment tool.

**Materials and methods:**

The WHO measles programmatic risk assessment tool was used to estimate the overall risk of measles in 31 providences and 322 districts of Iran at 2017. The measles risk was calculated by a function of four indicator scores including population immunity, surveillance quality, program performance, and threat assessment and the overall risk of measles for each districts calculated. Then, the tool assigned each district a risk category of low, medium, high, or very high according to the overall risk score.

**Results:**

Of the 322 districts in Iran, all districts were categorized as low risk and there was no very high risk, high risk and medium risk district in Iran. Twenty-six districts (7.4%) received to risk point higher than 20. Based on population immunity and program delivery performance indicators, all districts in Iarn were categorized as low risk and 92.86% of districts were in low risk category by surveillance quality indicator.

**Conclusion:**

The overall risk of measles profile was categorized as low risk and Iranian practices for measles elimination is very good in comparing other studies in this area. However, more attempts should be conducted to sustaining the surveillance quality indicators in all districts.

## 1 Introduction

Based on the World Health Organization (WHO) reports the EMRO countries should decrease to more than 90% in complication of measles, 95% decrease in mortality from measles, and eradication of measles to 2010 [1, 2]. To prioritize efforts to strengthen implementation of elimination strategies, the Centers for Disease Control and Prevention and WHO developed a measles programmatic risk assessment tool to identify high-risk districts and guide and strengthen program activities at the subnational level [3, 4, 5, 6].

Decreasing in morbidity and mortality of measles in recent years is due to high immunization coverage and increase in surveillance quality [7, 8]. The overall coverage of full immunization in Iran for all routine vaccines is 96.8% [8] and the immunization coverage for MMR1 and MMR2 in under 5 year old children in suburb area of big Iranian cities was calculated as 97.1% and 94.9% respectively [7, 8]. Moreover, the Supplementary Immunization Activities (SIAs) in Iranian target population children was estimated 98.7% [9]. Despite global improvement in annual measles incidence and mortality since 2000, progress toward elimination goals has slowed. The World Health Organization (WHO) European Region (EUR) established a regional goal for measles and rubella elimination by 2015 [4]. The elimination target for measles in East Mediterranean Region (EMR) that Iran is located, was planned for 2010 and extended to 2020. Nevertheless, according to some documents Iran is near to elimination [10, 11, 12]. Nevertheless, the inadequacy and delay of vaccination in migrant population was higher [13]. In addition, based on regression models, delay in MMR vaccination in children is associated with city of living (faring from capital) and nationality [7, 14].

Some studies conducted recently in developing countries by the World Health Organization Measles Programmatic Risk Assessment Tool in Romania, Namibia and Philippines [3, 4, 5, 15]. These studies showed that Kriss et al study showed that 64% of districts in Romania, 32% in Namibia and 48% in the Philippines were categorized as very high or high risk. Risk Assessment Tool could be used to guide measles elimination strategies and to identify programmatic areas that require strengthening [3]. Regular annual measles programmatic risk assessments can be used to help plan risk mitigation activities and measure progress toward measles elimination [15]. Moreover, risk assessment results can be used as a guide for monitoring and supervision and conducting of nationwide SIA and in target area [9, 15]. Annual assessments using the programmatic risk tool could provide valuable information for immunization program and surveillance staff at the national level and in each district to guide activities to enhance measles elimination efforts, such as strengthening routine immunization services, improving immunization campaign planning, and intensifying surveillance [4]. This study aimed to estimate the risk of measles outbreak in different districts of Iran and to identify high-risk areas in order to guide measles elimination program activities based on WHO measles programmatic risk assessment tool.

## 2 Materials and methods

### 2.1 Setting and design

A cross sectional study conducted on data of measles during 2014–2016 between March to May 2017, the WHO measles programmatic risk assessment tool was used to estimate the overall risk of measles in different districts of Iran in order to guide and strengthen measles elimination program activities and reduce the risk of outbreaks. This tool assesses subnational programmatic risk as the sum of indicator scores in four categories including population immunity, surveillance quality, program performance, and threat assessment. Each District in a country is assigned to a programmatic risk category of low, medium, high, or very high risk based on the overall risk score. Scoring for each indicator score was developed based on expert consensus [6, 16]. Risk points were assessed based on the World Health Organization Measles Programmatic Risk Assessment methods that developed by Lam et al. [5]. The overall risk of measles for each districts calculated by a function of combined indicator scores and summing of four indicators [5]. Then, according to the overall score, the tool assigned each district a risk category of low, medium, high, or very high.

This tool is validated and used in other recent researches by US CDC and WHO [3, 4, 15]. As it is described in the methodological works by Lam et al study [5] and continued by others [3, 4, 15], the total possible points for each indicators is determined and calculated based on some indexes. First, population immunity has the highest risk point equal to 40. This point risk calculates according to the measles susceptibility using administrative vaccination coverage data for MCV1 and MCV2 and coverage achieved during measles supplemental immunization activities (SIAs) conducted within the past 3 years. This indicator also includes the proportion of suspected measles cases with unknown vaccination status or who were unvaccinated. Second, surveillance quality has total point risk 20 and assesses the district' ability to detect and confirm cases rapidly and accurately. These indicators include the non-measles discarded rate; the proportion of suspected measles cases with adequate investigation (investigation within 48 hours of notification and inclusion of 10 core variables); the proportion of cases with adequate specimen collection (within 28 days of rash onset); and the proportion of cases for whom laboratory results were available in a timely manner. Third, program performance has total possible points 16. This indicator evaluates specific aspects of routine immunization services including MCV1 and MCV2 coverage trend between three recent years, dropout rates from MCV1 to MCV2 and from first dose of diphtheria, pertussis, and tetanus vaccine (DPT1) to MCV1 based on administrative vaccination coverage data. Fourth, threat assessment has total possible points equal 16 and assesses the influence risk factors for measles virus exposure and transmission in the population. The indicators include reported measles cases among specific age groups, recent measles cases reported in a bordering District, population density, and presence of vulnerable groups.

### 2.2 Data collection

Case based surveillance data, population size, immunization coverage and knowledge about existing for vulnerable population groups were data of this research. In addition, the shape file of country based on each districts should be prepared. The required data inputs include readily-available and routinely collected data from the immunization and surveillance programs were prepared by the Center for Communicable Diseases Control (CCDC) in Ministry of Health and Medical Education (MOHME) of Iran. The four data input categories would be collected based on the reports in CCDC in MOHME Iran. These data were gathered previously based on health system surveillance and health research. We were collected the vulnerable groups data by an expert team in each districts. Presence of vulnerable population groups was assessed by local knowledge of EPI manager at the national or district level. Eight factors including 1)Presence of migrant population, internally displaced population, slums, or tribal communities 2) Resistant to vaccination (i.e., religious, cultural issues, etc.) 3) Security and safety concerns 4) Frequented by calamities/disasters 5) Poor access to health services due to terrain/transportation issues 6) Lack of local political support 7) Presence of high-traffic transportation hubs/major roads or bordering large urban areas (within and across countries) 8) Presence of areas with mass gatherings (i.e., trade/commerce, fairs, markets, sporting events, high density of tourists) was assessed and give one risk point to each district if exist any of vulnerable population group.

### 2.3 Data analysis

The risk score was calculated based on World Health Organization Measles Programmatic Risk Assessment Tool for each providence and Iran. In the next step, we compared all scores and risk category of districts. Data were analyzed in WHO Measles Programmatic Risk Assessment Tool that works under Excel and Geographic Information System (GIS) software. We used a later version of shape file for Iran that contains 322 districts because the new updated shape file for all districts of Iran did not found. Therefore, some new districts (cities) that are separated recently, are merged with the prior districts.

## 3 Results

**Fig. 1:**
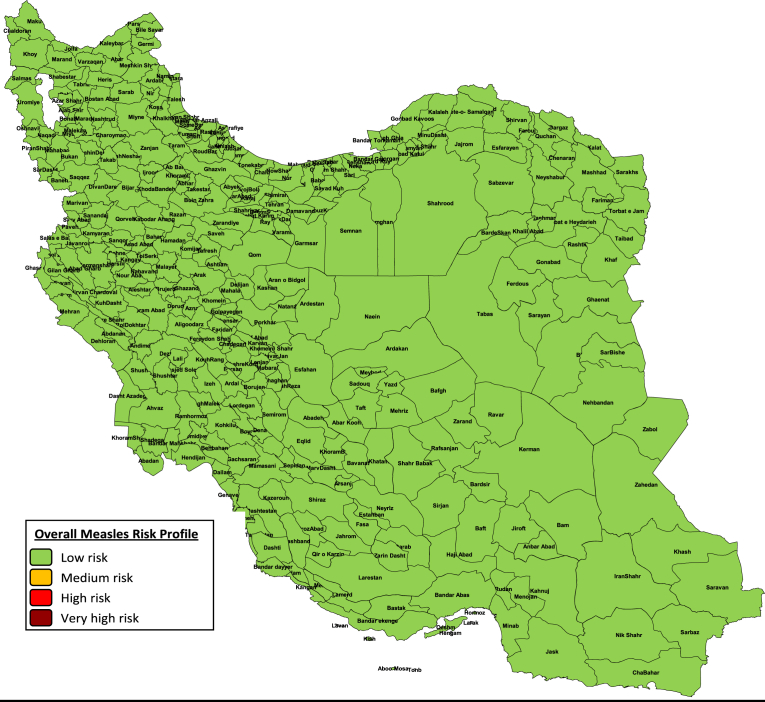
Overall measles risk assessment of Iran, 2014–2016.

This assessment conducted in Iran with 31 providences and 322 districts based on data of measles indictors in four items including population immunity, surveillance quality, program performance and threat assessment. Of the 322 Districts in Iran, 0 (0.0%) were categorized as very high risk, 0 (0.0%) were categorized as high risk, 0 (0.0%) were categorized as medium risk, and 322 (100.0%) were categorized as low risk. Therefore, according to Fig. 1, all Iranian districts categorized in low risk category based on Measles Risk Assessment, 2014–2016. We did not observed any district in medium, high risk and very high risk category in Iran.

Based on the overall risk points (Table 1), Saveh in Markazi, Mohr and Qir o Karzin in Fars provinces have the highest risk point in Iranian districts as 34, 30 and 29, respectively. However, 26 districts (7.4%) received to risk point higher than 20. The overall risk points for different districts in all provinces is showed at appendix 1.

**Table 1:** Overall Measles Risk Profile by districts with higher than 20 risk point in Measles risk assessment tool.

Area	Total risk points	Risk status	Population immunity	Surveillance quality	Program delivery performance	Threat assessment
Bushehr						
Jam	27	LR	6	20	0	1
East Azarbayjan						
Varzaqan	25	LR	0	20	2	3
Fars						
Arsanjan	24	LR	6	12	0	6
Eqlid	28	LR	0	20	4	4
Mohr	30	LR	6	20	0	4
Qir o Karzin	29	LR	0	20	4	5
Sepidan	20	LR	6	0	4	10
Gilan						
Masal	23	LR	0	20	2	1
Rasht	24	LR	0	16	0	8
Golestan						
Hormozgan						
Aboo Mosa	15	LR	0	12	0	3
Bandar Abas	18	LR	6	4	0	8
Bastak	23	LR	6	12	2	3
Haji Abad	26	LR	0	20	2	4
Hormoz	19	LR	0	12	4	3
Kish	24	LR	6	12	2	4
Isfahan						
Natanz	21	LR	6	12	2	1
Kerman						
Ravar	20	LR	0	20	0	0
Khuzestan						
Andimeshk	25	LR	6	12	4	3
Bandar Mahshahr	22	LR	6	0	2	14
Lali	21	LR	6	12	0	3
Markazi						
Saveh	34	LR	6	20	4	4
West.Azarbayjan						
PiranShahr	21	LR	0	20	0	1
Yazd						
Khatam	20	LR	6	12	2	0
Mehriz	22	LR	0	20	0	2
Zanjan						
Soltanieh	26	LR	0	20	2	4

According to our analysis about population immunity indicator, all districts in Iarn were categorized as low risk. The MCV1 and MCV2 coverage and their average in all Iranian districts was equal or higher 80% at 2014–2016. The coverage of MCV1 and MCV2 in all districts was higher 94% and the average 2014–2016 was higher 95%.

**Fig. 2:**
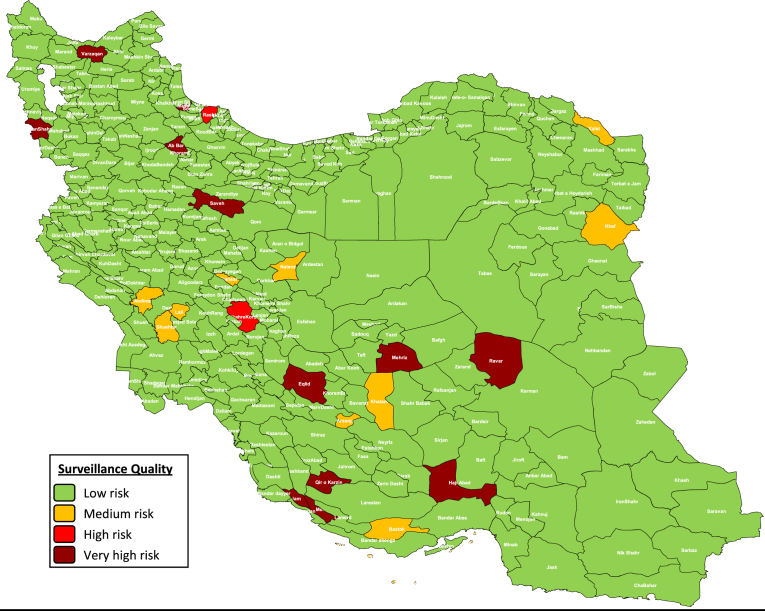
Mapping and categorization of Iranian districts based on the surveillance quality risk point.

The surveillance quality indicator (Fig. 2) in most districts (92.86%) of Iran is categorized in low risk. However, 9 districts (2.8%) were in Medium risk category. 2 districts (0.62%) categorized as high risk and 12 districts (3.73%) were defined as very high risk category.

Including Jam in Bushehr, Varzaqan in Esat.Azarbayjan, Eqlid, Mohr and Qir o Karzin in Fars, Masal in Gilan, Haji Abad in Hormozgan, Ravar in Kerman, Saveh in Markazi, PiranShahr in West Azarbayjan, Mehriz in Yazd and Soltanieh in Zanjan provinces.

**Fig. 3:**
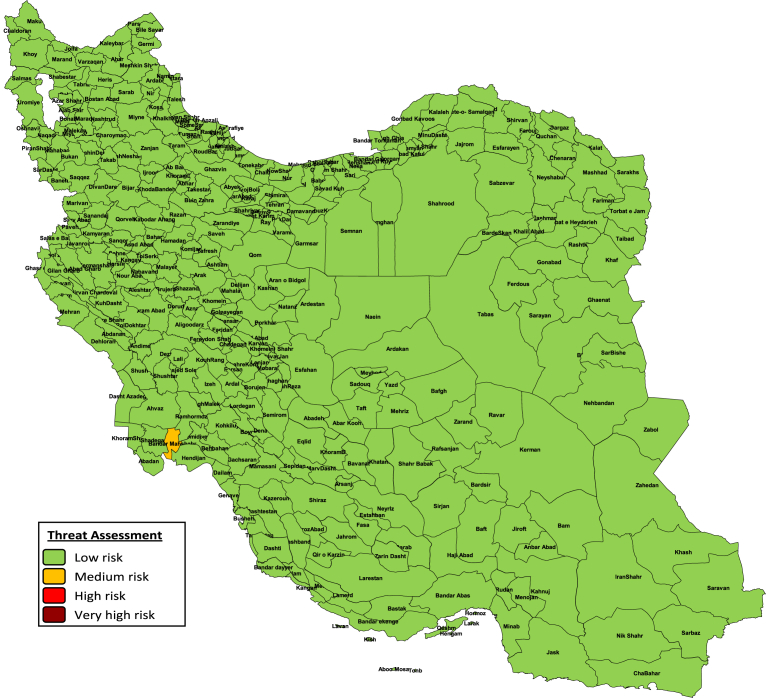
Mapping and categorization of Iranian districts based on the threat assessment risk point.

Based on program delivery performance indicator, all districts in Iran were categorized as low risk area. We have not any district in Iran that received Full Risk Points for all program delivery indicators. Based on our results, all districts in Iran have Drop-out Rate in MCV1-MCV2 and DPT1-MCV1 lower than 10%. Moreover, all districts in Iran were categorized as low risk based on threat assessment indicator (Fig. 3) except in Bandar mahshahr in Khuzestan province that categorized as medium risk.

The total number of measles cases was 815 at the study period. Therefore, the average incidence rate was 3.438 per one million people in Iran during 2014–2016. In addition, the immunization coverage for all districts was higher than 95% for MMR1 and MMR2.

## 4 Discussion

The results in our study prepared in 4 different sections including overall measles risk profile, population immunity, surveillance quality and program delivery according to the Measles Risk Assessment tool. The risk profile in 322 districts of Iran showed that all of them categorized as low risk 322 (100.0%) were categorized as low risk category. However, the status of Iran in control practices for elimination of measles is very good in comparing other studies in this area. In Kriss et al study, of the 42 districts in Romania, 27 (64%) were categorized as very high or high risk. The measles risk assessment conducted in Romania was the first assessment to be completed in a European country [4]. Another study based on the 2009 Measles Outbreak in Namibia conducted by Kriss et al and showed that of the 34 health districts in Namibia, 32% were classified as high risk or very high risk, including the district of Engela where the outbreak began in 2009 [3]. In another study in Philippines by 122 districts, 48% were classified as high risk or very high risk [15].

The high immunization coverage in all cities and villages of Iran is one of the most important factors of the low risk point of measles based the WHO risk assessment tool [7, 8]. Based on the risk points, Saveh in Markazi, Mohr and Qir o Karzin in Fars provinces have the highest risk point in Iranian districts and 7.4% of all districts received to risk point higher than 20. It is means that more attempt in these districts especially in surveillance quality and increase the adequate discard investigation cases.

The very high risk districts were more cities in border areas of Iran in all direction. But the southern regions were more in high risk category. In addition, Saveh in Markazi province is located at Southwest of Tehran that crowded by Afghanian immigrants. Another study showed that from 221 laboratory-confirmed measles cases during 2004–2009 in Iran, the most portion of cases were from rural areas and immigrant groups from high-incidence countries [17]. Therefore, supplementary immunization of children before starting in school in deprived and outskirt area suggested as an effective practice for decreasing the measles outbreaks risk [18] to protects the susceptible individuals in some areas who have not high enough immunity [19].

The increases in overall risk in districts with higher risk was as a result of poor surveillance quality primarily and in the second priority due to poor program performance and at the third level the including vulnerable population groups. Nevertheless, the population immunity in all districts of Iran was optimal. A same study that was the first assessment in a European country by Kriss et al showed that many of the very-high-risk districts were clustered in the western part of the country or were clustered around the capital Bucharest in the southeastern part of the country. The overall risk scores in the very-high-risk districts were driven primarily by poor surveillance quality and suboptimal population immunity [4]. Another study by Kriss et al in Namibia showed that the district of Windhoek, had the highest overall risk score-driven primarily by poor population immunity and immunization program performance-and one of the highest incidences during the outbreak [3].

According to our results, the trend in MCV1 and MCV2 coverage and high coverage of immunization beside the minimum of dropout rates from MCV1 to MCV2 and dropout rates from MCV1 to first dose of DPT0 or Pentavalent vaccine are important factors for low overall risk of measles in Iran. Recent studies in Iran showed that there are some documentations for elimination of measles [7, 9, 10, 11, 18, 20]. The subnational coverage of measles and supplemental immunization activities (SIAs) for target age group should be done annually in high risk and very high risk districts that have poor surveillance quality. However, based on recent research in Iran this index was 98.7% [9]. Rapid and wide progress regarding to elimination and eradication of measles was conducted in Iran [11]. Karami et al study showed that The Effective Reproduction Number (R) value of measles in Iran in 2012 was 0.87 and this index decrease to 0.76 at 2014 [10]. According to a study by Izadi et al, the seroprevalence rates of antibodies against measles in lower 16 age year Iranian children was 98.4% [20]. Nevertheless, continuous efforts must be made to improve and maintain the surveillance quality indicators especially in non-measles discarded rate, the proportion of suspected measles cases with adequate investigation, the ability of a district to detect and confirm cases with available laboratory results and the proportion of cases with adequate specimen collection indicators should in a desirable situation. Moreover, cold chain management did not considered in estimation of measles risk as a part of the risk assessment tool. Therefore, the performance quality of cold chain of vaccination could be added as a potential modification to the tool.

## 5 Conclusion

The overall risk of measles profile was acceptable in all districts of Iran and all districts were categorized as low risk. In addition, the average population immunity in all districts of Iran was very high. Therefore, the status of Iran in control practices for elimination of measles is very good in comparing other studies in this area. Therefore, the overall coverage of MCV1 and MCV2 and DPT should be kept high as well as minimum dropout rate. However, more attempts should be conducted to all districts have the surveillance quality indicators higher than WHO expectations.

## Declarations

### Author contribution statement

Abolfazl Mohammadbeigi: Conceived and designed the experiments; Analyzed and interpreted the data; Contributed reagents, materials, analysis tools or data; Wrote the paper.

Seyed Mohsen Zahraei: Conceived and designed the experiments; Contributed reagents, materials, analysis tools or data; Wrote the paper.

Azadeh Asgarian, Sima Afrashteh, Narges Mohammadsalehi, Salman Khazaei, Hossein Ansari: Analyzed and interpreted the data; Contributed reagents, materials, analysis tools or data; Wrote the paper.

### Funding statement

This work was supported by the WHO office in Iran collaboration Center.

### Competing interest statement

The authors declare no conflict of interest.

### Additional information

No additional information is available for this paper.
