# Supplementary material for: Estimation of measles risk using the World Health Organization Measles Programmatic Risk Assessment Tool, Iran
Source: Heliyon. 2018 Nov 1;4(11):e00886. doi: 10.1016/j.heliyon.2018.e00886 (PMC6218648; doi:10.1016/j.heliyon.2018.e00886)
Supplement: Appendix 1 [file mmc1.docx]

**Appendix 1: Risk profile by each districts and province**

| ***AREA*** | ***TOTAL RISK POINTS (100)*** | ***RISK STATUS*** | ***POPULATION IMMUNITY (40)*** | ***SURVEILLANCE QUALITY (20)*** | ***PROGRAM DELIVERY PERFORMANCE (16)*** | ***THREAT ASSESSMENT (24)*** |
| --- | --- | --- | --- | --- | --- | --- |
| *Alborz* |  |  |  |  |  |  |
| *Karaj* | ***6*** | **LR** | ***0*** | ***0*** | ***0*** | ***6*** |
| *NazarAbad* | ***7*** | **LR** | ***0*** | ***0*** | ***2*** | ***5*** |
| *SavojBolaq* | ***10*** | **LR** | ***0*** | ***0*** | ***2*** | ***8*** |
| *Ardabil* |  |  |  |  |  |  |
| *Ardabil* | ***6*** | **LR** | ***0*** | ***0*** | ***4*** | ***2*** |
| *Bile Savar* | ***4*** | **LR** | ***0*** | ***0*** | ***0*** | ***4*** |
| *Germi* | ***6*** | **LR** | ***0*** | ***0*** | ***4*** | ***2*** |
| *Khalkhal* | ***6*** | **LR** | ***0*** | ***0*** | ***4*** | ***2*** |
| *Kosar* | ***3*** | **LR** | ***0*** | ***0*** | ***2*** | ***1*** |
| *Meshkin Shahr* | ***4*** | **LR** | ***0*** | ***0*** | ***4*** | ***0*** |
| *Namin* | ***4*** | **LR** | ***0*** | ***0*** | ***2*** | ***2*** |
| *Nir* | ***2*** | **LR** | ***0*** | ***0*** | ***2*** | ***0*** |
| *Pars Abad* | ***10*** | **LR** | ***0*** | ***0*** | ***4*** | ***6*** |
| *Bushehr* |  |  |  |  |  |  |
| *Bandar dayyer* | ***0*** | **LR** | ***0*** | ***0*** | ***0*** | ***0*** |
| *Bushehr* | ***3*** | **LR** | ***0*** | ***0*** | ***0*** | ***3*** |
| *Dailam* | ***12*** | **LR** | ***0*** | ***8*** | ***2*** | ***2*** |
| *Dashtestan* | ***5*** | **LR** | ***0*** | ***4*** | ***0*** | ***1*** |
| *Dashti* | ***0*** | **LR** | ***0*** | ***0*** | ***0*** | ***0*** |
| *Genaveh* | ***5*** | **LR** | ***0*** | ***0*** | ***4*** | ***1*** |
| *Jam* | ***27*** | **LR** | ***6*** | ***20*** | ***0*** | ***1*** |
| *Kangan* | ***2*** | **LR** | ***0*** | ***0*** | ***0*** | ***2*** |
| *Tangestan* | ***0*** | **LR** | ***0*** | ***0*** | ***0*** | ***0*** |
| *Charmahal* |  |  |  |  |  |  |
| *Ardal* | ***2*** | **LR** | ***0*** | ***0*** | ***2*** | ***0*** |
| *Borujen* | ***2*** | **LR** | ***0*** | ***0*** | ***2*** | ***0*** |
| *Farsan* | ***4*** | **LR** | ***0*** | ***0*** | ***2*** | ***2*** |
| *KouhRang* | ***8*** | **LR** | ***6*** | ***0*** | ***0*** | ***2*** |
| *Lordegan* | ***11*** | **LR** | ***0*** | ***8*** | ***2*** | ***1*** |
| *ShahreKord* | ***19*** | **LR** | ***0*** | ***16*** | ***0*** | ***3*** |
| *Esat.Azarbayjan* |  |  |  |  |  |  |
| *Ahar* | ***11*** | **LR** | ***6*** | ***0*** | ***4*** | ***1*** |
| *Ajab Shir* | ***1*** | **LR** | ***0*** | ***0*** | ***0*** | ***1*** |
| *Azar Shahr* | ***4*** | **LR** | ***0*** | ***0*** | ***2*** | ***2*** |
| *Bonab* | ***5*** | **LR** | ***0*** | ***0*** | ***2*** | ***3*** |
| *Bostan Abad* | ***7*** | **LR** | ***0*** | ***0*** | ***4*** | ***3*** |
| *Charoymaq* | ***7*** | **LR** | ***0*** | ***0*** | ***4*** | ***3*** |
| *Hashtrud* | ***9*** | **LR** | ***0*** | ***0*** | ***4*** | ***5*** |
| *Heris* | ***5*** | **LR** | ***0*** | ***0*** | ***4*** | ***1*** |
| *Jolfa* | ***3*** | **LR** | ***0*** | ***0*** | ***0*** | ***3*** |
| *Kaleybar* | ***0*** | **LR** | ***0*** | ***0*** | ***0*** | ***0*** |
| *Malekan* | ***5*** | **LR** | ***0*** | ***0*** | ***0*** | ***5*** |
| *Marand* | ***4*** | **LR** | ***0*** | ***0*** | ***0*** | ***4*** |
| *Maraqe* | ***7*** | **LR** | ***0*** | ***0*** | ***0*** | ***7*** |
| *Miyne* | ***9*** | **LR** | ***0*** | ***0*** | ***4*** | ***5*** |
| *Osku* | ***10*** | **LR** | ***0*** | ***4*** | ***4*** | ***2*** |
| *Sarab* | ***6*** | **LR** | ***0*** | ***0*** | ***4*** | ***2*** |
| *Shabestar* | ***15*** | **LR** | ***0*** | ***8*** | ***4*** | ***3*** |
| *Tabriz* | ***7*** | **LR** | ***0*** | ***0*** | ***2*** | ***5*** |
| *Varzaqan* | ***25*** | **LR** | ***0*** | ***20*** | ***2*** | ***3*** |
| *Fars* |  |  |  |  |  |  |
| *Abadeh* | ***13*** | **LR** | ***6*** | ***0*** | ***0*** | ***7*** |
| *Arsanjan]* | ***24*** | **LR** | ***6*** | ***12*** | ***0*** | ***6*** |
| *Bavanat* | ***3*** | **LR** | ***0*** | ***0*** | ***0*** | ***3*** |
| *Darab* | ***9*** | **LR** | ***0*** | ***4*** | ***0*** | ***5*** |
| *Eqlid* | ***28*** | **LR** | ***0*** | ***20*** | ***4*** | ***4*** |
| *Estahban* | ***7*** | **LR** | ***0*** | ***4*** | ***0*** | ***3*** |
| *Farashband* | ***3*** | **LR** | ***0*** | ***0*** | ***0*** | ***3*** |
| *Fasa* | ***5*** | **LR** | ***0*** | ***0*** | ***2*** | ***3*** |
| *FirozAbad* | ***13*** | **LR** | ***0*** | ***8*** | ***2*** | ***3*** |
| *Jahrom* | ***9*** | **LR** | ***0*** | ***0*** | ***2*** | ***7*** |
| *Kazeroun* | ***3*** | **LR** | ***0*** | ***0*** | ***0*** | ***3*** |
| *KhoramBid* | ***4*** | **LR** | ***0*** | ***0*** | ***2*** | ***2*** |
| *Lamerd* | ***2*** | **LR** | ***0*** | ***0*** | ***0*** | ***2*** |
| *Larestan* | ***9*** | **LR** | ***0*** | ***0*** | ***0*** | ***9*** |
| *Mamasani* | ***8*** | **LR** | ***0*** | ***4*** | ***0*** | ***4*** |
| *MarvDasht* | ***5*** | **LR** | ***0*** | ***0*** | ***0*** | ***5*** |
| *Mohr* | ***30*** | **LR** | ***6*** | ***20*** | ***0*** | ***4*** |
| *NeyrIz* | ***4*** | **LR** | ***0*** | ***0*** | ***0*** | ***4*** |
| *Qir o Karzin* | ***29*** | **LR** | ***0*** | ***20*** | ***4*** | ***5*** |
| *Sepidan* | ***20*** | **LR** | ***6*** | ***0*** | ***4*** | ***10*** |
| *Shiraz* | ***17*** | **LR** | ***0*** | ***8*** | ***0*** | ***9*** |
| *Zarin Dasht* | ***4*** | **LR** | ***0*** | ***0*** | ***0*** | ***4*** |
| *Gilan* |  |  |  |  |  |  |
| *Amlash* | ***5*** | **LR** | ***0*** | ***0*** | ***2*** | ***3*** |
| *Astaneh Ashrafiye* | ***5*** | **LR** | ***0*** | ***0*** | ***0*** | ***5*** |
| *Astara* | ***5*** | **LR** | ***0*** | ***0*** | ***0*** | ***5*** |
| *Bandar Anzali* | ***6*** | **LR** | ***0*** | ***0*** | ***0*** | ***6*** |
| *Fuman* | ***6*** | **LR** | ***0*** | ***4*** | ***0*** | ***2*** |
| *Lahijan* | ***10*** | **LR** | ***0*** | ***4*** | ***0*** | ***6*** |
| *Langrud* | ***16*** | **LR** | ***0*** | ***8*** | ***4*** | ***4*** |
| *Masal* | ***23*** | **LR** | ***0*** | ***20*** | ***2*** | ***1*** |
| *Rasht* | ***24*** | **LR** | ***0*** | ***16*** | ***0*** | ***8*** |
| *Rezvan Shahr* | ***1*** | **LR** | ***0*** | ***0*** | ***0*** | ***1*** |
| *RoudBar* | ***6*** | **LR** | ***0*** | ***0*** | ***4*** | ***2*** |
| *RudSar* | ***8*** | **LR** | ***0*** | ***4*** | ***0*** | ***4*** |
| *Shaft* | ***5*** | **LR** | ***0*** | ***0*** | ***2*** | ***3*** |
| *SiahKal* | ***16*** | **LR** | ***10*** | ***0*** | ***4*** | ***2*** |
| *SomeSara* | ***4*** | **LR** | ***0*** | ***0*** | ***0*** | ***4*** |
| *Talesh* | ***9*** | **LR** | ***0*** | ***8*** | ***0*** | ***1*** |
| *Golestan* |  |  |  |  |  |  |
| *Agh Ghla* | ***3*** | **LR** | ***0*** | ***0*** | ***0*** | ***3*** |
| *Ali Abad Katul* | ***8*** | **LR** | ***6*** | ***0*** | ***0*** | ***2*** |
| *Azad Shahr* | ***4*** | **LR** | ***0*** | ***0*** | ***0*** | ***4*** |
| *Bandar Gaz* | ***5*** | **LR** | ***0*** | ***0*** | ***0*** | ***5*** |
| *Bandar Torkaman* | ***17*** | **LR** | ***0*** | ***8*** | ***4*** | ***5*** |
| *Gonbad Kavoos* | ***2*** | **LR** | ***0*** | ***0*** | ***0*** | ***2*** |
| *Gorgan* | ***9*** | **LR** | ***0*** | ***4*** | ***0*** | ***5*** |
| *Kalaleh* | ***1*** | **LR** | ***0*** | ***0*** | ***0*** | ***1*** |
| *Kord Kuy* | ***6*** | **LR** | ***0*** | ***0*** | ***0*** | ***6*** |
| *MinuDasht* | ***3*** | **LR** | ***0*** | ***0*** | ***0*** | ***3*** |
| *Ramyan* | ***6*** | **LR** | ***0*** | ***0*** | ***2*** | ***4*** |
| *Hamedan* |  |  |  |  |  |  |
| *Asad Abad* | ***7*** | **LR** | ***0*** | ***0*** | ***4*** | ***3*** |
| *Bahar* | ***7*** | **LR** | ***0*** | ***0*** | ***4*** | ***3*** |
| *Hamadan* | ***10*** | **LR** | ***0*** | ***4*** | ***0*** | ***6*** |
| *Kabodar Ahang* | ***5*** | **LR** | ***0*** | ***0*** | ***2*** | ***3*** |
| *Malayer* | ***9*** | **LR** | ***0*** | ***4*** | ***4*** | ***1*** |
| *Nahavand* | ***5*** | **LR** | ***0*** | ***0*** | ***0*** | ***5*** |
| *Razan* | ***5*** | **LR** | ***0*** | ***0*** | ***4*** | ***1*** |
| *ToiSerkan* | ***8*** | **LR** | ***0*** | ***0*** | ***4*** | ***4*** |
| *Hormozgan* |  |  |  |  |  |  |
| *Aboo Mosa* | ***15*** | **LR** | ***0*** | ***12*** | ***0*** | ***3*** |
| *Bandar Abas* | ***18*** | **LR** | ***6*** | ***4*** | ***0*** | ***8*** |
| *Bandar Lengeh* | ***7*** | **LR** | ***0*** | ***4*** | ***0*** | ***3*** |
| *Bastak* | ***23*** | **LR** | ***6*** | ***12*** | ***2*** | ***3*** |
| *Haji Abad* | ***26*** | **LR** | ***0*** | ***20*** | ***2*** | ***4*** |
| *Hormoz* | ***19*** | **LR** | ***0*** | ***12*** | ***4*** | ***3*** |
| *Jask* | ***11*** | **LR** | ***0*** | ***4*** | ***0*** | ***7*** |
| *Kish* | ***24*** | **LR** | ***6*** | ***12*** | ***2*** | ***4*** |
| *Minab* | ***15*** | **LR** | ***6*** | ***0*** | ***0*** | ***9*** |
| *Qeshm* | ***17*** | **LR** | ***6*** | ***4*** | ***0*** | ***7*** |
| *Rudan* | ***6*** | **LR** | ***0*** | ***0*** | ***2*** | ***4*** |
| *Ilam* |  |  |  |  |  |  |
| *Abdanan* | ***7*** | **LR** | ***6*** | ***0*** | ***0*** | ***1*** |
| *Dare Shahr* | ***7*** | **LR** | ***0*** | ***0*** | ***4*** | ***3*** |
| *Dehloran* | ***9*** | **LR** | ***6*** | ***0*** | ***0*** | ***3*** |
| *Eyvan* | ***5*** | **LR** | ***0*** | ***0*** | ***2*** | ***3*** |
| *Ilam* | ***10*** | **LR** | ***6*** | ***0*** | ***0*** | ***4*** |
| *Mehran* | ***6*** | **LR** | ***6*** | ***0*** | ***0*** | ***0*** |
| *Shirvan Chardoval* | ***11*** | **LR** | ***0*** | ***4*** | ***4*** | ***3*** |
| *Isfahan* |  |  |  |  |  |  |
| *Aran o Bidgol* | ***10*** | **LR** | ***0*** | ***4*** | ***2*** | ***4*** |
| *Ardestan* | ***4*** | **LR** | ***0*** | ***0*** | ***0*** | ***4*** |
| *Borkhar* | ***3*** | **LR** | ***0*** | ***0*** | ***0*** | ***3*** |
| *Chadegan* | ***8*** | **LR** | ***0*** | ***0*** | ***4*** | ***4*** |
| *Dehaghan* | ***11*** | **LR** | ***6*** | ***0*** | ***4*** | ***1*** |
| *Esfahan* | ***12*** | **LR** | ***6*** | ***0*** | ***0*** | ***6*** |
| *FalavarJan* | ***4*** | **LR** | ***0*** | ***0*** | ***0*** | ***4*** |
| *Faridan* | ***3*** | **LR** | ***0*** | ***0*** | ***0*** | ***3*** |
| *Fereydon Shahr* | ***1*** | **LR** | ***0*** | ***0*** | ***0*** | ***1*** |
| *Golpayegan* | ***6*** | **LR** | ***0*** | ***0*** | ***0*** | ***6*** |
| *Kashan* | ***5*** | **LR** | ***0*** | ***0*** | ***0*** | ***5*** |
| *Khansar* | ***17*** | **LR** | ***0*** | ***12*** | ***4*** | ***1*** |
| *Khomeini Shahr* | ***5*** | **LR** | ***0*** | ***0*** | ***0*** | ***5*** |
| *Lenjan* | ***9*** | **LR** | ***0*** | ***0*** | ***4*** | ***5*** |
| *Mobarakeh* | ***4*** | **LR** | ***0*** | ***0*** | ***2*** | ***2*** |
| *Naein* | ***2*** | **LR** | ***0*** | ***0*** | ***0*** | ***2*** |
| *Najaf Abad* | ***13*** | **LR** | ***0*** | ***4*** | ***4*** | ***5*** |
| *Natanz* | ***21*** | **LR** | ***6*** | ***12*** | ***2*** | ***1*** |
| *Semirom* | ***3*** | **LR** | ***0*** | ***0*** | ***0*** | ***3*** |
| *ShahReza* | ***4*** | **LR** | ***0*** | ***0*** | ***0*** | ***4*** |
| *Tiran o Karvan* | ***4*** | **LR** | ***0*** | ***0*** | ***0*** | ***4*** |
| *Kerman* |  |  |  |  |  |  |
| *Anbar Abad* | ***3*** | **LR** | ***0*** | ***0*** | ***0*** | ***3*** |
| *Baft* | ***6*** | **LR** | ***0*** | ***0*** | ***2*** | ***4*** |
| *Bam* | ***17*** | **LR** | ***0*** | ***8*** | ***0*** | ***9*** |
| *Bardsir* | ***7*** | **LR** | ***0*** | ***0*** | ***4*** | ***3*** |
| *Jiroft* | ***17*** | **LR** | ***6*** | ***8*** | ***0*** | ***3*** |
| *Kahnuj* | ***13*** | **LR** | ***6*** | ***0*** | ***4*** | ***3*** |
| *Kerman* | ***8*** | **LR** | ***6*** | ***0*** | ***0*** | ***2*** |
| *Menojan* | ***2*** | **LR** | ***0*** | ***0*** | ***0*** | ***2*** |
| *Rafsanjan* | ***4*** | **LR** | ***0*** | ***0*** | ***2*** | ***2*** |
| *Ravar* | ***20*** | **LR** | ***0*** | ***20*** | ***0*** | ***0*** |
| *Shahr Babak* | ***4*** | **LR** | ***0*** | ***0*** | ***0*** | ***4*** |
| *Sirjan* | ***9*** | **LR** | ***6*** | ***0*** | ***0*** | ***3*** |
| *Zarand* | ***1*** | **LR** | ***0*** | ***0*** | ***0*** | ***1*** |
| *Kermanshah* |  |  |  |  |  |  |
| *Eslam Abad Gharb* | ***2*** | **LR** | ***0*** | ***0*** | ***0*** | ***2*** |
| *Ghasr e Shirin* | ***1*** | **LR** | ***0*** | ***0*** | ***0*** | ***1*** |
| *Gilan Gharb* | ***7*** | **LR** | ***6*** | ***0*** | ***0*** | ***1*** |
| *Harsin* | ***5*** | **LR** | ***0*** | ***0*** | ***2*** | ***3*** |
| *Javanroud* | ***1*** | **LR** | ***0*** | ***0*** | ***0*** | ***1*** |
| *Kangavar* | ***9*** | **LR** | ***0*** | ***0*** | ***2*** | ***7*** |
| *Kermanshah* | ***3*** | **LR** | ***0*** | ***0*** | ***0*** | ***3*** |
| *Paveh* | ***6*** | **LR** | ***0*** | ***0*** | ***4*** | ***2*** |
| *Sahne* | ***4*** | **LR** | ***0*** | ***0*** | ***0*** | ***4*** |
| *Salas e Babjani* | ***6*** | **LR** | ***0*** | ***0*** | ***4*** | ***2*** |
| *Sanqor* | ***4*** | **LR** | ***0*** | ***0*** | ***0*** | ***4*** |
| *Sarpol Zahab* | ***2*** | **LR** | ***0*** | ***0*** | ***0*** | ***2*** |
| *Khorasan.Jonobi* |  |  |  |  |  |  |
| *Birjand* | ***2*** | **LR** | ***0*** | ***0*** | ***0*** | ***2*** |
| *Nehbandan* | ***8*** | **LR** | ***6*** | ***0*** | ***0*** | ***2*** |
| *Sarayan* | ***0*** | **LR** | ***0*** | ***0*** | ***0*** | ***0*** |
| *SarBishe* | ***10*** | **LR** | ***0*** | ***4*** | ***4*** | ***2*** |
| *Khorasan.Razavi* |  |  |  |  |  |  |
| *BardeSkan* | ***4*** | **LR** | ***0*** | ***0*** | ***2*** | ***2*** |
| *Chenaran* | ***3*** | **LR** | ***0*** | ***0*** | ***0*** | ***3*** |
| *Dargaz* | ***4*** | **LR** | ***0*** | ***0*** | ***2*** | ***2*** |
| *Fariman* | ***7*** | **LR** | ***0*** | ***4*** | ***0*** | ***3*** |
| *Ferdous* | ***2*** | **LR** | ***0*** | ***0*** | ***0*** | ***2*** |
| *Ghaenat* | ***5*** | **LR** | ***0*** | ***0*** | ***4*** | ***1*** |
| *Gonabad* | ***2*** | **LR** | ***0*** | ***0*** | ***2*** | ***0*** |
| *Kalat* | ***12*** | **LR** | ***0*** | ***12*** | ***0*** | ***0*** |
| *Kashmar* | ***4*** | **LR** | ***0*** | ***0*** | ***2*** | ***2*** |
| *Khaf* | ***12*** | **LR** | ***0*** | ***12*** | ***0*** | ***0*** |
| *Khalil Abad* | ***8*** | **LR** | ***0*** | ***8*** | ***0*** | ***0*** |
| *Mashhad* | ***8*** | **LR** | ***0*** | ***0*** | ***0*** | ***8*** |
| *Neyshabur* | ***7*** | **LR** | ***0*** | ***0*** | ***4*** | ***3*** |
| *Quchan* | ***0*** | **LR** | ***0*** | ***0*** | ***0*** | ***0*** |
| *Rashtkhar* | ***2*** | **LR** | ***0*** | ***0*** | ***0*** | ***2*** |
| *Sabzevar* | ***12*** | **LR** | ***6*** | ***4*** | ***0*** | ***2*** |
| *Sarakhs* | ***3*** | **LR** | ***0*** | ***0*** | ***0*** | ***3*** |
| *Taibad* | ***3*** | **LR** | ***0*** | ***0*** | ***0*** | ***3*** |
| *Torbat e Heydarieh* | ***4*** | **LR** | ***0*** | ***0*** | ***0*** | ***4*** |
| *Torbat e Jam* | ***15*** | **LR** | ***6*** | ***0*** | ***4*** | ***5*** |
| *Khorasan.Shomali* |  |  |  |  |  |  |
| *Bojnourd* | ***13*** | **LR** | ***6*** | ***0*** | ***4*** | ***3*** |
| *Esfarayen* | ***8*** | **LR** | ***0*** | ***8*** | ***0*** | ***0*** |
| *Farouj* | ***6*** | **LR** | ***0*** | ***4*** | ***2*** | ***0*** |
| *Jajrom* | ***2*** | **LR** | ***0*** | ***0*** | ***0*** | ***2*** |
| *Mete-o- Samalqan* | ***2*** | **LR** | ***0*** | ***0*** | ***0*** | ***2*** |
| *Shirvan* | ***0*** | **LR** | ***0*** | ***0*** | ***0*** | ***0*** |
| *Khuzestan* |  |  |  |  |  |  |
| *Abadan* | ***10*** | **LR** | ***0*** | ***8*** | ***0*** | ***2*** |
| *Ahvaz* | ***8*** | **LR** | ***0*** | ***0*** | ***4*** | ***4*** |
| *Andimeshk* | ***25*** | **LR** | ***6*** | ***12*** | ***4*** | ***3*** |
| *BaghMalek* | ***2*** | **LR** | ***0*** | ***0*** | ***2*** | ***0*** |
| *Bandar Mahshahr* | ***22*** | **LR** | ***6*** | ***0*** | ***2*** | ***14*** |
| *Behbahan* | ***3*** | **LR** | ***0*** | ***0*** | ***0*** | ***3*** |
| *Dasht Azadegan* | ***0*** | **LR** | ***0*** | ***0*** | ***0*** | ***0*** |
| *Dezful* | ***9*** | **LR** | ***0*** | ***0*** | ***4*** | ***5*** |
| *Hendijan* | ***10*** | **LR** | ***0*** | ***0*** | ***4*** | ***6*** |
| *Izeh* | ***6*** | **LR** | ***0*** | ***4*** | ***0*** | ***2*** |
| *KhoramShahr* | ***17*** | **LR** | ***6*** | ***8*** | ***0*** | ***3*** |
| *Lali* | ***21*** | **LR** | ***6*** | ***12*** | ***0*** | ***3*** |
| *Masjed Soleyman* | ***5*** | **LR** | ***0*** | ***0*** | ***2*** | ***3*** |
| *Omidiye* | ***18*** | **LR** | ***0*** | ***4*** | ***4*** | ***10*** |
| *Ramhormoz* | ***5*** | **LR** | ***0*** | ***0*** | ***2*** | ***3*** |
| *Shadegan* | ***9*** | **LR** | ***6*** | ***0*** | ***0*** | ***3*** |
| *Shush* | ***8*** | **LR** | ***0*** | ***0*** | ***4*** | ***4*** |
| *Shushtar* | ***16*** | **LR** | ***0*** | ***12*** | ***0*** | ***4*** |
| *Kohkiloye* |  |  |  |  |  |  |
| *BoyerAhmad* | ***4*** | **LR** | ***0*** | ***0*** | ***0*** | ***4*** |
| *Dena* | ***11*** | **LR** | ***6*** | ***0*** | ***4*** | ***1*** |
| *Gachsaran* | ***11*** | **LR** | ***6*** | ***0*** | ***2*** | ***3*** |
| *Kohkiluyeh* | ***3*** | **LR** | ***0*** | ***0*** | ***0*** | ***3*** |
| *Kordestan* |  |  |  |  |  |  |
| *Baneh* | ***8*** | **LR** | ***0*** | ***4*** | ***0*** | ***4*** |
| *Bijar* | ***8*** | **LR** | ***6*** | ***0*** | ***0*** | ***2*** |
| *DivanDare* | ***2*** | **LR** | ***0*** | ***0*** | ***0*** | ***2*** |
| *Kamyaran* | ***1*** | **LR** | ***0*** | ***0*** | ***0*** | ***1*** |
| *Marivan* | ***1*** | **LR** | ***0*** | ***0*** | ***0*** | ***1*** |
| *Qorveh* | ***5*** | **LR** | ***0*** | ***0*** | ***2*** | ***3*** |
| *Sanandaj* | ***3*** | **LR** | ***0*** | ***0*** | ***0*** | ***3*** |
| *Saqqez* | ***5*** | **LR** | ***0*** | ***0*** | ***2*** | ***3*** |
| *Sarv Abad* | ***0*** | **LR** | ***0*** | ***0*** | ***0*** | ***0*** |
| *Lorestan* |  |  |  |  |  |  |
| *Aleshtar* | ***4*** | **LR** | ***0*** | ***0*** | ***2*** | ***2*** |
| *Aligoodarz* | ***4*** | **LR** | ***0*** | ***0*** | ***0*** | ***4*** |
| *Azna* | ***5*** | **LR** | ***0*** | ***0*** | ***2*** | ***3*** |
| *Brujerd* | ***3*** | **LR** | ***0*** | ***0*** | ***0*** | ***3*** |
| *Dorud* | ***6*** | **LR** | ***0*** | ***0*** | ***2*** | ***4*** |
| *Khoram Abad* | ***5*** | **LR** | ***0*** | ***0*** | ***2*** | ***3*** |
| *KuhDasht* | ***4*** | **LR** | ***0*** | ***0*** | ***0*** | ***4*** |
| *Nour Abad* | ***3*** | **LR** | ***0*** | ***0*** | ***0*** | ***3*** |
| *PolDokhtar* | ***8*** | **LR** | ***0*** | ***8*** | ***0*** | ***0*** |
| *Markazi* |  |  |  |  |  |  |
| *Arak* | ***9*** | **LR** | ***0*** | ***4*** | ***2*** | ***3*** |
| *Ashtian* | ***10*** | **LR** | ***6*** | ***0*** | ***0*** | ***4*** |
| *Delijan* | ***2*** | **LR** | ***0*** | ***0*** | ***0*** | ***2*** |
| *Khomein* | ***4*** | **LR** | ***0*** | ***0*** | ***4*** | ***0*** |
| *Komijan* | ***5*** | **LR** | ***0*** | ***0*** | ***2*** | ***3*** |
| *Mahalat* | ***3*** | **LR** | ***0*** | ***0*** | ***2*** | ***1*** |
| *Saveh* | ***34*** | **LR** | ***6*** | ***20*** | ***4*** | ***4*** |
| *Shazand* | ***4*** | **LR** | ***0*** | ***4*** | ***0*** | ***0*** |
| *Tafresh* | ***3*** | **LR** | ***0*** | ***0*** | ***0*** | ***3*** |
| *Zarandiye* | ***2*** | **LR** | ***0*** | ***0*** | ***0*** | ***2*** |
| *Mazandaran* |  |  |  |  |  |  |
| *Amol* | ***5*** | **LR** | ***0*** | ***0*** | ***2*** | ***3*** |
| *Babol* | ***3*** | **LR** | ***0*** | ***0*** | ***2*** | ***1*** |
| *BabolSar* | ***5*** | **LR** | ***0*** | ***0*** | ***4*** | ***1*** |
| *Behshahr* | ***9*** | **LR** | ***0*** | ***0*** | ***0*** | ***9*** |
| *Chalus* | ***13*** | **LR** | ***0*** | ***4*** | ***0*** | ***9*** |
| *Ghaem Shahr* | ***4*** | **LR** | ***0*** | ***4*** | ***0*** | ***0*** |
| *Juybar* | ***13*** | **LR** | ***0*** | ***8*** | ***4*** | ***1*** |
| *Mahmud Abad* | ***5*** | **LR** | ***0*** | ***4*** | ***0*** | ***1*** |
| *Neka* | ***7*** | **LR** | ***0*** | ***0*** | ***2*** | ***5*** |
| *NowShahr* | ***4*** | **LR** | ***0*** | ***0*** | ***0*** | ***4*** |
| *Nur* | ***8*** | **LR** | ***0*** | ***4*** | ***0*** | ***4*** |
| *Ramsar* | ***2*** | **LR** | ***0*** | ***0*** | ***0*** | ***2*** |
| *Sari* | ***2*** | **LR** | ***0*** | ***0*** | ***0*** | ***2*** |
| *Savad Kuh* | ***1*** | **LR** | ***0*** | ***0*** | ***0*** | ***1*** |
| *Tonekabon* | ***9*** | **LR** | ***0*** | ***0*** | ***0*** | ***9*** |
| *Qazvin* |  |  |  |  |  |  |
| *Abyek* | ***3*** | **LR** | ***0*** | ***0*** | ***0*** | ***3*** |
| *Buin Zahra* | ***4*** | **LR** | ***0*** | ***0*** | ***0*** | ***4*** |
| *Ghazvin* | ***10*** | **LR** | ***0*** | ***0*** | ***0*** | ***10*** |
| *Takestan* | ***5*** | **LR** | ***0*** | ***0*** | ***0*** | ***5*** |
| *Qom* |  |  |  |  |  |  |
| *Qom* | ***9*** | **LR** | ***0*** | ***0*** | ***0*** | ***9*** |
| *Semnan* |  |  |  |  |  |  |
| *Damghan* | ***7*** | **LR** | ***0*** | ***0*** | ***2*** | ***5*** |
| *Garmsar* | ***8*** | **LR** | ***0*** | ***0*** | ***2*** | ***6*** |
| *Semnan* | ***5*** | **LR** | ***0*** | ***0*** | ***0*** | ***5*** |
| *Shahrood* | ***8*** | **LR** | ***6*** | ***0*** | ***0*** | ***2*** |
| *Sistan* |  |  |  |  |  |  |
| *ChaBahar* | ***10*** | **LR** | ***0*** | ***0*** | ***0*** | ***10*** |
| *IranShahr* | ***3*** | **LR** | ***0*** | ***0*** | ***0*** | ***3*** |
| *Khash* | ***9*** | **LR** | ***6*** | ***0*** | ***0*** | ***3*** |
| *Nik Shahr* | ***7*** | **LR** | ***0*** | ***4*** | ***0*** | ***3*** |
| *Saravan* | ***12*** | **LR** | ***6*** | ***0*** | ***0*** | ***6*** |
| *Sarbaz* | ***8*** | **LR** | ***6*** | ***0*** | ***0*** | ***2*** |
| *Zabol* | ***4*** | **LR** | ***0*** | ***4*** | ***0*** | ***0*** |
| *Zahedan* | ***9*** | **LR** | ***6*** | ***0*** | ***0*** | ***3*** |
| *Tehran* |  |  |  |  |  |  |
| *Damavand* | ***3*** | **LR** | ***0*** | ***0*** | ***0*** | ***3*** |
| *EslamShahr* | ***6*** | **LR** | ***0*** | ***0*** | ***0*** | ***6*** |
| *FirouzKuh* | ***8*** | **LR** | ***6*** | ***0*** | ***0*** | ***2*** |
| *PakDasht* | ***5*** | **LR** | ***0*** | ***0*** | ***0*** | ***5*** |
| *Ray* | ***4*** | **LR** | ***0*** | ***0*** | ***0*** | ***4*** |
| *Robat Karim* | ***9*** | **LR** | ***0*** | ***0*** | ***0*** | ***9*** |
| *Shahriyar* | ***11*** | **LR** | ***0*** | ***0*** | ***2*** | ***9*** |
| *Shimiranat* | ***8*** | **LR** | ***6*** | ***0*** | ***0*** | ***2*** |
| *Tehran* | ***14*** | **LR** | ***0*** | ***0*** | ***4*** | ***10*** |
| *Varamin* | ***10*** | **LR** | ***6*** | ***0*** | ***0*** | ***4*** |
| *West.Azarbayjan* |  |  |  |  |  |  |
| *Bukan* | ***6*** | **LR** | ***0*** | ***0*** | ***2*** | ***4*** |
| *Chaldoran* | ***4*** | **LR** | ***0*** | ***0*** | ***4*** | ***0*** |
| *Khoy* | ***9*** | **LR** | ***0*** | ***8*** | ***0*** | ***1*** |
| *Mahabad* | ***1*** | **LR** | ***0*** | ***0*** | ***0*** | ***1*** |
| *Maku* | ***0*** | **LR** | ***0*** | ***0*** | ***0*** | ***0*** |
| *MiyandoAb* | ***7*** | **LR** | ***0*** | ***0*** | ***4*** | ***3*** |
| *Naqade* | ***7*** | **LR** | ***0*** | ***0*** | ***2*** | ***5*** |
| *Oshnaviye* | ***11*** | **LR** | ***6*** | ***0*** | ***4*** | ***1*** |
| *PiranShahr* | ***21*** | **LR** | ***0*** | ***20*** | ***0*** | ***1*** |
| *Salmas* | ***10*** | **LR** | ***0*** | ***8*** | ***0*** | ***2*** |
| *SarDasht* | ***13*** | **LR** | ***0*** | ***8*** | ***4*** | ***1*** |
| *ShahinDej* | ***13*** | **LR** | ***0*** | ***8*** | ***0*** | ***5*** |
| *Takab* | ***4*** | **LR** | ***0*** | ***0*** | ***2*** | ***2*** |
| *Uromiye* | ***11*** | **LR** | ***0*** | ***8*** | ***0*** | ***3*** |
| *Yazd* |  |  |  |  |  |  |
| *Abar Kooh* | ***3*** | **LR** | ***0*** | ***0*** | ***0*** | ***3*** |
| *Ardakan* | ***2*** | **LR** | ***0*** | ***0*** | ***0*** | ***2*** |
| *Bafgh* | ***8*** | **LR** | ***6*** | ***0*** | ***0*** | ***2*** |
| *Khatam* | ***20*** | **LR** | ***6*** | ***12*** | ***2*** | ***0*** |
| *Mehriz* | ***22*** | **LR** | ***0*** | ***20*** | ***0*** | ***2*** |
| *Meybod* | ***6*** | **LR** | ***0*** | ***0*** | ***4*** | ***2*** |
| *Sadouq* | ***7*** | **LR** | ***6*** | ***0*** | ***0*** | ***1*** |
| *Tabas* | ***2*** | **LR** | ***0*** | ***0*** | ***2*** | ***0*** |
| *Taft* | ***2*** | **LR** | ***0*** | ***0*** | ***2*** | ***0*** |
| *Yazd* | ***8*** | **LR** | ***6*** | ***0*** | ***0*** | ***2*** |
| *Zanjan* |  |  |  |  |  |  |
| *Soltanieh* | ***26*** | **LR** | ***0*** | ***20*** | ***2*** | ***4*** |
| *Abhar* | ***4*** | **LR** | ***0*** | ***0*** | ***0*** | ***4*** |
| *Ijrood* | ***4*** | **LR** | ***0*** | ***0*** | ***4*** | ***0*** |
| *KhodaBandeh* | ***0*** | **LR** | ***0*** | ***0*** | ***0*** | ***0*** |
| *KhoramDareh* | ***12*** | **LR** | ***6*** | ***0*** | ***2*** | ***4*** |
| *MahNeshan* | ***2*** | **LR** | ***0*** | ***0*** | ***2*** | ***0*** |
| *Taram* | ***6*** | **LR** | ***0*** | ***0*** | ***4*** | ***2*** |
| *Zanjan* | ***3*** | **LR** | ***0*** | ***0*** | ***2*** | ***1*** |
